# Internet and smartphone-based ecological momentary assessment and personalized advice (PROfeel) in adolescents with chronic conditions: A feasibility study

**DOI:** 10.1016/j.invent.2021.100395

**Published:** 2021-04-20

**Authors:** Merel M. Nap-van der Vlist, Jan Houtveen, Geertje W. Dalmeijer, Martha A. Grootenhuis, Cornelis K. van der Ent, Martine van Grotel, Joost F. Swart, Joris M. van Montfrans, Elise M. van de Putte, Sanne L. Nijhof

**Affiliations:** a Department of Pediatrics, Wilhelmina Children's Hospital, University Medical Center Utrecht, Utrecht University, Utrecht, the Netherlands; b Julius Center for Health Sciences and Primary Care, University Medical Center Utrecht, Utrecht University, Utrecht, the Netherlands; c Princess Máxima Center for Pediatric Oncology, Utrecht, the Netherlands; d Department of Pediatric Pulmonology, Wilhelmina Children's Hospital, University Medical Center Utrecht, Utrecht University, Utrecht, the Netherlands; e Department of Pediatric Rheumatology/Immunology and Infectious Diseases, Wilhelmina Children's Hospital, University Medical Center Utrecht, Utrecht University, Utrecht, the Netherlands

**Keywords:** EMA, ecological momentary assessments, ILD, intensive longitudinal data, CF, cystic fibrosis, VAS, visual analogue scale, FEV_1_, forced expiratory volume in one second, JIA, juvenile idiopathic arthritis, cJADAS, clinical Juvenile Arthritis Disease Activity Score, ESR, erythrocyte sedimentation rate, SD, standard deviation, Ecological momentary assessments, Personalized feedback, Adolescents, Chronic disease, Fatigue

## Abstract

**Objective:**

Growing up with a chronic disease comes with challenges, such as coping with fatigue. Many adolescents are severely fatigued, though its associated factors exhibit considerable interpersonal and longitudinal variation. We assessed whether PROfeel, a combination of a smartphone-based ecological momentary assessment (EMA) method using the internet, followed by a face-to-face dialogue and personalized advice for improvement of symptoms or tailor treatment based on a dynamic network analysis report, was feasible and useful.

**Study design:**

Feasibility study in fatigued outpatient adolescents 12–18 years of age with cystic fibrosis, autoimmune disease, post-cancer treatment, or with medically unexplained fatigue. Participants were assessed at baseline to personalize EMA questions. EMA was conducted via smartphone notifications five times per day for approximately six weeks. Hereby, data was collected via the internet. The EMA results were translated into a personalized report, discussed with the participant, and subsequently translated into a personalized advice. Afterwards, semi-structured interviews on feasibility and usefulness were held.

**Results:**

Fifty-seven adolescents were assessed (mean age 16.2 y ± 1.6, 16% male). Adolescents deemed the smartphone-based EMA feasible, with the app being used for an average of 49 days. Forty-two percent of the notifications were answered and 85% of the participants would recommend the app to other adolescents. The personalized report was deemed useful and comprehensible and 95% recognized themselves in the personalized report, with 64% rating improved insight in their symptoms and subsequent steps towards an approach to reduce one's fatigue as good or very good.

**Conclusions:**

PROfeel was found to be highly feasible and useful for fatigued adolescents with a chronic condition. This innovative method has clinical relevance through bringing a patient's daily life into the clinical conversation.

## Highlights

• Fatigued adolescents with a chronic condition could use insight in their symptoms.
• Fatigue and associated factors were measured via ecological momentary assessments.
• Data were analyzed with dynamic network analyses, leading to personalized feedback.
• This combination of assessment and feedback (PROfeel), was feasible and useful.
• This innovative method brings a patient's daily life into the clinical conversation.

## 1 Introduction

### 1.1 Background

An ever increasing number of children are growing up with a chronic disease (Perrin et al., 2007). In the Netherlands, approximately one in four children is affected with a chronic disease (Verwey-Jonker Instituut, 2019). They face extra challenges associated with their ever-present illness, such as coping with fatigue or pain (Armbrust et al., 2015; Jóhannsdóttir et al., 2012; Nap-van der Vlist et al., 2019). Fatigue can be a debilitating symptom, leading to reduced societal participation and quality of life (Huang et al., 2013; Kim et al., 2014; Nijhof et al., 2016). Although various factors may play a role in fatigue in childhood chronic disease, the exact predisposing, precipitating, and perpetuating roles differ on an individual level (interpersonal) and over time (longitudinal) (Ebner-Priemer and Trull, 2009; Frijns et al., 2020; Kratz et al., 2017; Molenaar, 2004). To tailor advice to the unique adolescent's needs and mechanisms in a way that fits the adolescent's lifestyle and perception, new personalized (internet and smartphone-based) longitudinal assessment methodologies and data analysis strategies are needed (Hamaker and Wichers, 2017).

### 1.2 Conceptualization of fatigue

Fatigue could be considered a transdiagnostic symptom (Menting et al., 2018; Nap-van der Vlist et al., 2019). This means that various factors that perpetuate fatigue may be shared by adolescents with a chronic disease, as they may be part of growing up with a chronic disease, independent of their diagnosis (Stein and Jessop, 1989). Examples of such biological, psychological and social factors that influence outcome measures in pediatric chronic diseases (Armbrust et al., 2016; Maurice-Stam et al., 2019; Nap-van der Vlist et al., 2019), include sleep patterns, physical activity, and psychological wellbeing. Recent studies show that disease-specific factors may play a role in perpetuating fatigue, but fatigue may persist despite low or absent disease activity (Van Dijk-Lokkart et al., 2019; Nijhof et al., 2016). Therefore, potentially modifiable factors other than disease-specific factors may be associated with – and can perpetuate – fatigue in these patients and are potential treatment targets on an individual level (Armbrust et al., 2016; Deary et al., 2007; Engel, 1977; Langeveld et al., 2003; Nap-van der Vlist et al., 2018; Nijhof et al., 2016; Vijver et al., 2019).

### 1.3 Ecological momentary assessment and single-subject data analyses

To find out which modifiable factors are associated with fatigue in daily life on an individual level, it is possible to collect highly intensive longitudinal data (ILD) of participants. Real-time fluctuations in one's symptoms and well-being can be investigated through micro-questionnaires multiple times per day (Stone and Shiffman, 1994). This Ecological Momentary Assessment method (EMA) can nowadays be implemented using the internet and smartphones to reliably collect more than 50 assessments per person (Myin-Germeys et al., 2009; van Roekel et al., 2019). With increasing computational power, it has recently become feasible to perform dynamic ILD-analyses (Hamaker and Wichers, 2017). Using the combination of EMA with novel data analysis techniques such as dynamic structural equation modelling (DSEM; (Asparouhov et al., 2018)), it is possible to gain patient-tailored insights into underlying mechanisms of symptoms such as fatigue, when measured with EMA (Houtveen et al., 2015; Houtveen and Sorbi, 2013; Kroeze et al., 2017; Myin-Germeys et al., 2009; Van Roekel et al., 2017). The application of this concept into clinically applicable tools, especially in pediatric medicine, is innovative. Examples of feasible applications are yet limited, taking into account the high intensity of the longitudinal measurements and the applicability of personalized feedback (Bell et al., 2017; Kramer et al., 2014; Kroeze et al., 2017; Van Roekel et al., 2017; van Roekel et al., 2019).

It may be useful for fatigued adolescents with a chronic condition to combine internet-based EMA and ILD analyses to identify, for a particular patient, the core biopsychosocial processes that are related to fatigue in the individual patient (Hofmann and Hayes, 2019). These dynamic processes and patterns can be used to start a dialogue within the adolescent-clinician relationship on how the adolescent can self-manage his/her fatigue, formulating personalized advice for improvement of symptoms (e.g., by lifestyle changes) or to tailor a subsequent treatment. In this study, we will refer to the combination of this measurement method and personalized advice as PROfeel. In the current study, we assessed whether PROfeel is feasible and useful. We defined feasibility as achievability of filling out the EMA measurements and understanding the personalized report. We defined usefulness as effective in helping adolescents gain insight in their symptoms and associated factors.

## 2 Material and methods

### 2.1 Study design

Feasibility study among outpatients of the Wilhelmina Children's Hospital and the Princess Máxima Center for Pediatric Oncology in the Netherlands, from October 2017 through August 2018. Using patient-reported outcomes (PROs), we assessed the feasibility and usefulness of PROfeel, a combination of EMA (using the internet and smartphones) followed by a personalized advice based on a personalized report. We defined feasibility as achievability of filling out the EMA measurements (Feasibility EMA) and understanding the personalized report (Feasibility Report). For the EMA, we looked at compliance (Feasibility EMA 1), user friendliness (Feasibility EMA 2) and the burden of the measurements for adolescents (Feasibility EMA 3). For the personalized report, we looked at whether adolescents found it recognizable (Feasibility Report 1) and comprehensible (Feasibility Report 2). We defined usefulness as effective in helping adolescents gain insight in their symptoms and associated factors. For the EMA, we looked at whether adolescents found it valuable to fill out the EMA and whether they would recommend it to other adolescents (Usefulness EMA). For the personalized report, we asked adolescents to report on new insight based on the report (Usefulness Report 1) and whether they were able to take steps towards an approach to reduce one's fatigue (Usefulness Report 2). This study was classified by the institutional review board as exempt of the Medical Research Involving Human Subjects Act (16-203/C). Informed consent was obtained from adolescents and their parents.

### 2.2 Participants

Participants were recruited from a larger cohort of children with cystic fibrosis (CF), an autoimmune disease, children post-cancer treatment or children with medically unexplained symptoms, at the Wilhelmina Children's Hospital and the Princess Máxima Center for Pediatric Oncology in the Netherlands: the PROactive cohort. The goal of the PROactive cohort is to collect data on fatigue and associated factors across childhood chronic diseases, in a relatively stable phase of their disease. The methodology of data collection of the PROactive cohort is described elsewhere (Nap-van der Vlist et al., 2019). Included in this study were adolescents 12–18 years of age with cystic fibrosis (CF), autoimmune disease, post-cancer treatment, or with medically unexplained fatigue. Participants were eligible if their treating physician determined they were in a stable phase of their disease, if they scored fatigued (see below) on a screening questionnaire of the PROactive data collection and if they indicated that this fatigue restricted them in daily life. For the screening on fatigue, the Dutch version of the PedsQL multidimensional fatigue scale was used (Gordijn et al., 2011). A cut-off of more than one standard deviation below the norm was taken to classify fatigued adolescents, taking into account sex and age (Nap-van der Vlist et al., 2019). This norm was chosen in order to be able to offer PROfeel to a broad range of adolescents. The most important inclusion criteria were whether the adolescent him/herself felt restricted by his/her fatigue in daily life, actively desired to gain insight into his/her symptoms and had a wish to actively work on reducing his/her fatigue.

### 2.3 Content of personalized ecological momentary assessments

For this feasibility study, an existing EMA-platform and application was used that allowed for the delivery of personalized EMA prompts (notifications) and questions on the smartphone, namely the Ethica application (Ethica Data, 2019). Part of the questions of the EMA-survey was the same for all participants (fixed) and part was personalized, based on personal preference. The EMA content was built on the theoretical framework of the efficacious elements of cognitive behavioral therapy for fatigue in adolescents (Nijhof et al., 2012). Central elements in this therapy are, for example, establishing a sleep routine, stimulating an adolescent to find a balance between rest and activity, gradually increasing activity at home, school or with friends, addressing fatigue-related cognitions, and increasing self-management and independency (Nijhof et al. 2011). Therefore, the fixed content of the EMA questionnaire (i.e., collected for each participant) included questions regarding sleep-wake routines, physical activity and social activity. The personalized part of the EMA questionnaire was focused on targeting a participant's individual cognitions of fatigue (see Supplementary Table S1 for an overview of the EMA content). The content was developed in an interdisciplinary team of researchers and clinicians from the fields of psychology, pediatrics, information technology, and graphic design. This was an iterative process in which potential questions were reviewed and tested several times by team members, after which the content and usefulness was tested by a patient.

There were 13 fixed questions. Fatigue and restrictions were measured on a visual analogue scale (VAS) from 0 to 100, and school participation was measured by asking for amount of hours the participant attended school compared to the amount of scheduled hours. In addition, fixed questions were nighttime and daytime sleep, physical activity and mental activity (e.g. working concentrated on a task or paying attention), and social support. These factors were measured as VAS scores (0–100) or categorical outcomes (two to four answer options).

The personalized questions were chosen based on a baseline survey with the participant. Up to three other physical complaints could be chosen that seemed to be associated with fatigue for this particular participant. Furthermore, associated personal factors in thinking or feeling (psychosocial factors) or personal factors that could be associated with the perceived symptoms in the broadest sense of the word could be chosen. Up to five personal factors could be added in total. For example, one participant wanted to add a variable about his food intake, and another requested to add a variable about her boyfriend and the pressure she felt while being with him. Little has been reported in the literature on the ideal length of EMA questionnaires (van Roekel et al., 2019). The choice for the number of EMA questions requires balancing between collecting as much data as possible, while minimizing the burden for adolescents who participated. To achieve minimal burden, we chose to partially personalize the EMA questionnaires, and branching was used to tailor some questions to the specific situation (i.e. other questions were asked in the morning then in the afternoon). Attention was paid to the length of the total EMA survey, so that it did not take more than one minute to fill out per notification. The maximum of questions was therefore set to 26. The selection of questions was implemented by filling out a baseline survey on the smartphone during intake (see below).

### 2.4 Study procedures

If an adolescent was willing to participate, the researcher planned an intake to install the app and discuss the personalization of the EMA. Next, the participant downloaded the application on his/her smartphone and filled out the baseline survey, leading to the personalization of both the EMA prompts (adjusted to the waking hours of the participant) and the EMA content. After completion, the personalized EMA started. A signal-contingent method was chosen, in which participants received notifications on their smartphone approximately five times per day, every three hours during waking hours. Data were transferred to a server using the internet, but the EMA could also be filled out when there was no internet connection. Results were then uploaded when the smartphone was reconnected to the internet. A time interval of three hours was chosen, since it was hypothesized that this interval would be suitable to measure changes in fatigue, although empirical evidence in this field is still lacking (Dormann and Griffin, 2015; Hamaker and Wichers, 2017). Participants were followed for approximately six weeks. In order to optimize the ILD analyses, we aimed for at least 50–100 measurements per participant and at least three measurements per day (Voelkle et al., 2012). This means that more measurements were offered than adolescents needed to fill out, allowing the adolescent to miss measurements occasionally, for example when they were busy or in class. After the measurement period, a personalized report was drafted by a statistician/psychologist and the clinical researcher (see Section 2.5 and Table 4). This report was discussed by the clinical researcher with the participant and, if possible, with their physician or psychologist. The goal of this appointment was to let the adolescent discover his or her own data, ask questions and help him/her gain insight in one's own symptoms so he/she could, together with the clinical researcher and/or healthcare provider, formulate a personalized advice for improvement of symptoms or to tailor treatment. After the discussion of the personalized report, a semi-structured interview with the participant on feasibility and usefulness was performed by another researcher who was not present at the discussion of the personalized report, to avoid socially desirable answers.

### 2.5 Personalized report

#### 2.5.1 Analyzing the EMA data

Several ways were used to gain insight in the intensive longitudinal data. Two report methods were used to gain insight in the intensive longitudinal data: a basic report method and a RDSEM-based report. For the basic report, we used descriptive statistics. For the RDSEM report, we used innovative ILD dynamic time series analyses showing the single-subject dynamics of a process over time, yielding Granger causality (Hamaker and Wichers, 2017). Granger causality means that a change in one concept repeatedly follows a change in another concept, also referred to as a lagged relationship or cross-correlation. For example, every time an adolescent scored higher on worrying, later that day this adolescent scored higher on fatigue. Granger causality, however, only suggests causality but does not proof it. We presented such a result to the adolescent as the hypothesis that fatigue follows worrying, and although we do not proof causality, it provides a clue. We used RDSEM-frameworks to create individual dynamic networks to identify complaint-perpetuating and influencing factors such as cognitions, affect, physical and mental activity, social support, and sleep quality. A more detailed description of the statistical background of these analyses can be found in Supplementary Table S2. RDSEM analyses were done in MPLUS version 8.2.

#### 2.5.2 Presenting the EMA data in a personalized report

The personalized report always started with a basic report with graphs showing the observed scores of the measured continuous variables (such as fatigue or pain, but also thoughts, feelings, amount of physical or mental activity, etc.). For each variable, the low-frequency trend (i.e., low-pass filtered signal) was also visualized in the same graph. The mean scores per hour of the day and per day of the week were shown in different graphs in the basic report to gain insight in the temporal patterns. Dependency of the continuous variables with the categorical factors observed (e.g., being alone or with others, being at home, at school, somewhere else, etc.) was visualized in the basic report by showing graphs with the mean scores per category. Using this basic method, adolescents could still gain insight in their symptoms, even when a RDSEM analyses was not possible, or yielded little or no clear, significant results. After the basic analyses, all continuous factors were entered into different RDSEM models (max three variables per model) to compute dynamic networks showing the lagged and contemporaneous relationships between these variables, corrected for the low-frequency trends, time of day and weekend effects. RDSEM dynamic networks that were considered of relevance were shown in simplified form to the participant. This was based on: 1) was an estimate significant with P < 0.05, 2) had an estimate a standardized β > 0.2, and 3) was an estimate considered clinically relevant. Examples of the visual representation of these networks are found in Table 4. This visual representation was developed and refined based on feedback from the interdisciplinary research team and participants.

### 2.6 Measures

#### 2.6.1 Clinical inclusion measurements

Patient characteristics such as age, sex, diagnosis, duration of disease and disease activity were extracted from the participant's medical record. For adolescents with CF, forced expiratory volume in one second (FEV_1_) was used as a proxy for disease status, expressed as the percentage of predicted FEV_1_ (Quanjer et al., 2012). For adolescents with juvenile idiopathic arthritis (JIA), the clinical Juvenile Arthritis Disease Activity Score (cJADAS) and erythrocyte sedimentation rate (ESR) were used as a proxy for disease status; for adolescents with other autoimmune diseases, only ESR was used.

#### 2.6.2 Compliance assessment

To assess compliance, the number of notifications sent and answered was recorded. Also, the number of days that EMA was used was recorded.

#### 2.6.3 Semi-structured interviews to assess feasibility and usefulness

The participants were asked to grade eight aspects of the use of the EMA and five aspects of the personalized report (scale of 10 points, questionnaire especially developed for this study). On all these aspects, the researcher encouraged the participant with open questions to express why the specific grade was given and what worked well on this aspect and what could further be improved.

### 2.7 Data analyses feasibility and usefulness

Descriptive statistics were used to summarize patient characteristics, compliance data and ratings as feedback on the different aspects of the EMA and the personalized report. Differences between participants and non-participants were analyzed using the Student's *t*-test or chi-square test. Normally distributed data are presented as mean ± standard deviation (SD); otherwise median and interquartile range was provided. A generic explorative qualitative design was used with a two-step approach. All interviews were transcribed and themes were coded and categorized (Holloway et al., 2010). Main themes were described and illustrated with quotes.

## 3 Results

### 3.1 Patient characteristics

Of the 97 approached adolescents, 57 (59%) participated. The mean age was 16.2 ± 1.6 years and 16% was male. There were ten participants with CF, 18 with an autoimmune disease or immune deficiency, three post-cancer treatment, and 26 with medically unexplained fatigue (Table 1). There were no differences in sex and age between the participants and non-participants, nor were there significant differences in participation rates between groups. Common reasons for not participating were finding it too burdensome to fill out the EMA or not wanting to be confronted with their fatigue. There was no significant difference in fatigue scores between participants and non-participants.

**Table 1:** Baseline characteristics of participants.

	Chronic disease groups			Adolescents with unexplained fatigue
	Adolescents with CF (N = 10)	Adolescents with autoimmune disease (N = 18)	Adolescents post-cancer treatment (N = 3)	Adolescents with medically unexplained fatigue (N = 26)
Age (mean ± SD)	16.9 ± 1.6	16.2 ± 1.8	15.1 ± 1.7	16.0 ± 1.4
Sex, female (N, %)	9 (90%)	16 (89%)	3 (100%)	20 (77%)
Diagnosis	4 (40%) homozygous dF508 mutation 6 (60%) heterozygous dF508 mutation	3 (17%) poly JIA 5 (28%) oligo JIA 2 (11%) systemic JIA 2 (11%) other JIA subtype 6 (33%) other autoimmune disease	1 (33%) solid tumors 2 (67%) leukemia/lymphoma	NA
Duration of disease, years^a^ (mean ± SD)	16.9 ± 1.6	5.2 ± 4.0	0.9 ± 0.8	NA
Disease activity^b^	FEV_1_%: 69.5 ± 15.1	cJADAS: 2 (0–9) ESR: 7.5 mm/1st hr (2–48)	All post-cancer treatment and in remission	NA

a Duration of disease: years since diagnosis until inclusion for adolescents with JIA; years from end of treatment until inclusion for adolescents post-cancer treatment.

b If the data were normally distributed, the mean ± SD is given; if not, the median and interquartile range is given.CF = cystic fibrosis; SD = standard deviation; JIA = juvenile idiopathic arthritis; cJADAS = clinical Juvenile Arthritis Disease Activity Score; ESR = erythrocyte sedimentation rate; FEV_1_% = predicted percentage of forced expiratory volume in one second; NA = not applicable.

### 3.2 Feasibility EMA measurements

#### 3.2.1 Feasibility EMA1: compliance

**Fig. 1:**
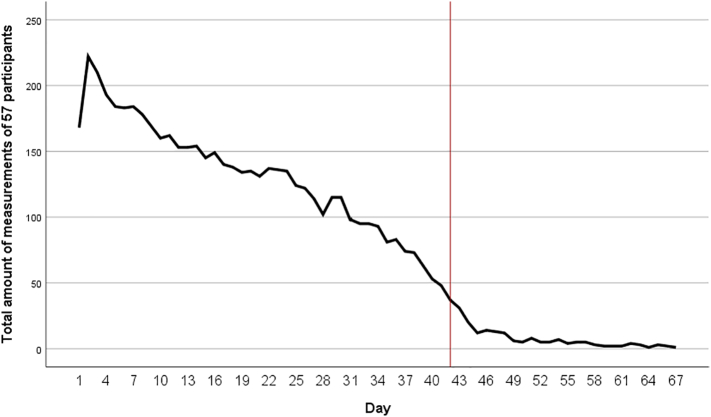
Total amount of EMA measurements in all participants per day. The red line indicates the desired measurement period of six weeks.

Participants received on average 236 ± 96 notifications with an average measurement period of 49 ± 22 (range 21–135) days. During this measurement period, participants filled out EMA questionnaires for an average of 36 ± 11 (range 10–67) days. Of all the notifications, 42% was answered. We strived for at least 50–100 measurements per participant. Ten participants (18%) did not reach this, due to technical difficulties, decreased motivation or a combination of these. Especially at the start of the study, some technical difficulties were encountered. One of the technical difficulties was that for IPhone users, the Ethica app generated duplicate data for expired surveys, which made it harder to reliably calculate the compliance rate. The reported compliance rate (42%) is the lowest calculated compliance rate. Compliance was highest at the start of the study and slowly decreased during the measurement period (Fig. 1).

#### 3.2.2 Feasibility EMA 2: user friendliness

All participants rated user friendliness as good or very good (Table 2). Of the 46 adolescents who commented on user friendliness, 42 (91%) found the EMA easy to fill out, short, clear and personal. *“The questions were really adapted to my situation and easy to relate to, so I immediately knew what to answer.” (Female, 17 years old).* Participants told us it took them between 40 s and five minutes to fill out and twelve participants mentioned that the time to fill out the EMA shortened during the measurement period.

**Table 2:** Usefulness and feasibility of the EMA.

Ranking	Clarity of EMA (n = 50)	User friendliness (n = 50)	Burden of filling out EMA during measurement period (n = 46)	Would recommend app (n = 47)
Very bad (0–2)	0	0	1 (2%)	2 (not at all; 4%)
Bad (3–4)	0	0	2 (4%)	0 (no)
Medium (5–6)	2 (4%)	0	10 (22%)	5 (maybe; 9%)
Good (7–8)	22 (44%)	20 (40%)	16 (35%)	16 (most likely; 34%)
Very good (9–10)	26 (52%)	30 (60%)	17 (37%)	24 (definitely; 51%)

Other positive points described by participants were the use of notifications on their own smartphone, the possibility to fill out the EMA with and without internet connection, and the use of visual analogue scales. The main barrier in user friendliness was the technical difficulties. Furthermore, almost halve of the adolescents (23 of the 49 that commented on this question, 47%) found it difficult that the EMA often expired when they were busy or in class. For three adolescents, the continuous confrontation with their symptoms was a negative experience. *“The amount of notifications was good, but the timing was bad. I did not always manage to fill out the EMA two or three times per day. When I felt bad, I was really tired, I did not sleep well and then I repeatedly had to fill out that I did not feel well.” (Female, 18 years old).*

#### 3.2.3 Feasibility EMA 3: burden of the measurement period

Thirty-eight of the 43 adolescents that commented on the length of the measurement period (88%) described the burden of the measurements as doable and part of their daily routine. For 17 of the 50 participants (34%) who commented on the amount of measurements per day, five notifications per day were too much and some described repeatedly filling out the same questions as annoying. Twenty-nine percent of the adolescents described the measurement period as long or too long, but most described that they found this period necessary to provide a clear overview of what their daily life looked like and only two of them found it too burdensome. Nine adolescents described that at the end of the measurement period, they were fed up with filling out the EMA and their motivation to continue to do it decreased during the measurement period. *“After four weeks I was tired of filling out the EMA, but I understand that you need six weeks for a good overview. It was not a bad thing, but irritating sometimes.” (Female, 18 years old).*

### 3.3 Feasibility of the personalized report

#### 3.3.1 Feasibility Report 1: recognizability

Most adolescents (96%) recognized their daily life patterns in the personalized report (that includes dynamic networks). Some adolescents did not recognize themselves in all associations and networks. One adolescent did not recognize her patterns at all and found the personalized report illogical. Recognition was needed for adolescents in order to be open to start a dialogue on personalized advice. For example, one adolescent told us: *“Here I recognized myself: sleeping while it does not really help. You stay tired.” (Female, 16 years old).* This insight based on her own data opened the conversation on sleep-wake rhythms and what she could do to improve them to alleviate her fatigue.

#### 3.3.2 Feasibility Report 2: comprehensibility

Comprehensibility was rated as good or very good by 86% of the participants. Twenty-four adolescents told us that the explanation of the personalized report and the conversation on its meaning was helpful. Two adolescents believed the personalized report would be clear enough with textual explanation, but most described the added value of the consultation with the researcher and healthcare provider as crucial. *“The graphs gave me insight and made me understand the results. I went to the appointment and said to my mom ‘I don't think anything came out of it [the app], because I don't feel it did.’ And then you have this conversation and it is really interesting, with all the graphs it becomes clearer.” (Female, 17 years old).*

### 3.4 Usefulness EMA

85% of the participants would recommend the smartphone-based EMA to other adolescents (Table 2). They found the EMA easy to fill out and not too time consuming. It helped them to think about their symptoms in another way. Nevertheless, they stated that discipline and time investment were required. *“At first I thought: what can this bring me? But I have really seen the result. I think other teenagers can benefit from this, an app that captures daily life and results in an overview: it could be this and this, so that you can take steps to deal with your symptoms.” (Female, 18 years old).* Two adolescents would not recommend PROfeel at all, because it did not generate new insights and it was seen as too much trouble.

### 3.5 Usefulness of the personalized report

#### 3.5.1 Usefulness Report 1: insight in symptoms

Of all participants, 64% reported insight in their symptoms. Results are shown in Table 3, with illustrative cases in Table 4. Adolescents valued the clear overview the personalized report gave them, a confirmation that the patterns they experienced were not just in their head, but could be made visible. Seventeen adolescents (35%) mentioned little or no new insights. The most common reason for this, given by nine adolescents, was because they were already aware of the patterns found in the personalized reports.

**Table 3:** Usefulness and feasibility of the personalized report.

Ranking	Recognition of personalized report (n = 44)	Comprehensibility of report (n = 42)	Insight in symptoms (n = 48)	Report enabled steps towards treatment (n = 40)
Very bad (0–2)	1 (2%)	1 (2%)	9 (19%)	8 (20%)
Bad (3–4)	0	0	1 (2%)	2 (5%)
Medium (5–6)	1 (2%)	5 (12%)	7 (15%)	5 (13%)
Good (7–8)	14 (32%)	11 (26%)	17 (35%)	15 (38%)
Very good (9–10)	28 (64%)	25 (60%)	14 (29%)	10 (25%)

**Table 4:** Examples of cases with quotes and graphs from the personalized report.

Case description	Quote	Example network technical report (results only shown when positive estimates (black) or negative estimates (red) > 0.1 and significant)	Example graph personalized report
1. 16 year old girl with medically unexplained back pain and fatigue. The personalized report shows more fatigue when mental activity is low. During the conversation we discovered that low mental inactivity for her meant also the absence of distraction. She concluded that distraction helps her to take her attention of her symptoms, which may help her. She was referred to a psychologist, with whom she is going to explore how she can use this knowledge to alleviate her symptoms.	*“That these things were not only my thoughts, buth there was actually a pattern.”*		
2. 17 year old girl with juvenile idiopathic arthritis. The personalized report shows a contemporaneous association between more fatigue and more mental activity and between more fatigue and more contact with people who bothered her or did not understand her. During our conversation she tells us that she often feels overstimulated in a busy environment by noises, emotions or talk. She concludes that she would be helped if someone could coach her on how to filter and regulate the incoming stimuli, so that they will cost her less energy.	*“Yes, now you look at it in another way. You notice for yourself when you’re tired, but you never write it down and now you suddenly see why you’re tired. So then you learn new things, you cannot record everything yourself, so this is nice: to know what makes you tired and what happens if you’re tired. That helped me anyway, this report. I know now precisely how it works.”*		
3. 14 year old girl with Hashimoto thyreoiditis. The personalized report shows that increased mental activity precedes more headache and more restrictions related to her symptoms, while being more physical activity precedes less headache and less restrictions. She tells us that she recognizes herself in this pattern, but she finds it really hard to motivate herself to get more physically active. She finds the possibility of cognitive behavioral therapy with graded exercise an option that may fit her needs.	*“It helps, because you know how you feel. If something comes up, it also helps the doctor. The doctor then sees how you’re doing over time and not just at one moment.”*		
4. 18 year old girl with prescleroderma. The personalized report shows that she has more pain in the weekends and when she is physically active. She tells us she intensively exercises in the weekend. She knows she will have more pain then, but accepts it in order to be able to play the hockey games. She does not want to change this. Furthermore, more worrying is followed in time by more fatigue. She tells us that she recognizes that her thinking can be really preoccupied every now and then. She did not make the link to fatigue before, but she is open to consider it as a possible treatment target.	*“Most of it I already knew. But you assess everything more thoroughly, so I got a little bit of new insight: that when I worry, it makes me feel more fatigued. That I feel worse after sports I knew, but also the confirmation is nice.”*		

#### 3.5.2 Usefulness Report II: steps towards an approach to reduce one's fatigue

Based on the personalized report, 63% reported they were able to take steps towards an approach to reduce one's fatigue (Table 3). Adolescents described the discussion of the report with the researcher and/or healthcare provider as valuable in taking steps towards an approach to reduce their fatigue. We will illustrate this with four example cases in Table 4, to show different outcomes of the EMA measurements and the discussion of the personalized report. In case 1, a contemporaneous association between mental inactivity and fatigue was found, leading to the collective conclusion that distraction (i.e., by being mentally active) may help this participant to take her mind of her symptoms. In contrast, in case 2, more mental activity was contemporaneously related with fatigue and prevention of overstimulation was defined as a possible treatment target. Case 3 shows us an example of how physical activity was followed (i.e., three hours later) by less symptoms (less headache in this case) and less restrictions. In case 4, however, physical activity in the weekends was associated with more symptoms. Also, in this latter case, worrying was followed by more fatigue. Regarding the tailored advice, in case 3, increasing physical activity may be the next step towards a tailored approach to reduce fatigue, while in case 4, psychological support to cope with worrying may be more effective in addition to more balanced physical activity during the weekend.

## 4 Discussion

### 4.1 Principal findings

This study describes the feasibility and usefulness of PROfeel, a combination of a smartphone-based EMA-method using the internet, followed by face-to-face dialogue and personalized advice for improvement of symptoms or to tailor treatment based on a dynamic network analysis report. Participants described the EMA as feasible and the personalized report and tailored advice as useful in gaining understanding in symptoms and taking steps towards an approach to reduce one's fatigue. Most participants would recommend this method (PROfeel) to other adolescents. There were large interindividual differences in the outcomes of the personalized reports and in the advices.

### 4.2 Feasibility and usefulness of EMA in adolescents with chronic conditions

In this feasibility study, we combined the feasibility and usefulness of EMA and a tailored advice based on a personalized report. EMA was already found feasible as a measurement instrument in participants with psychopathology and in several adolescent populations (Bell et al., 2017; van Roekel et al., 2019). In line with van Roekel et al., we found that a smartphone-based EMA-tool aligned with the adolescents' lifestyle was not an intrusive means of investigation, especially since adolescents became quicker in filling out the EMA during the measurement period and since EMA prompts were adjusted to their daily rhythm (van Roekel et al., 2019). Also, other electronic diary methods for children and adolescents with chronic diseases have been described before and were deemed feasible, but collection and storage of data keeps improving and opening new ways of collecting and analyzing increasing amounts of longitudinal data (i.e. (Bromberg et al., 2014; Connelly et al., n.d.; Elsbernd et al., 2018)). Some participants in our study described the continual confrontation while filling out EMA as a burden. Although burdensome, research suggests that when EMA is applied for a short period of time, this does not necessarily increase symptoms (Broderick and Vikingstad, 2008; Cruise et al., 1996; Van Roekel et al., 2017).

### 4.3 Feasibility and usefulness of the personalized report

Although a lot of applications to improve wellbeing for children and adolescents are available, evidence-based apps of good quality are scarce (Schoeppe et al., 2017). The application of personalized (i.e., patient-tailored) feedback based on single-subject EMA results is relatively new, but positive experiences in line with our study have already been described for young adults with anhedonia and depressed adults (Kramer et al., 2014; Kroeze et al., 2017; Van Roekel et al., 2017). Adolescents within our study described the use of PROfeel within the adolescent-clinician relationship in our study as valuable, in line with other studies describing an added value of personalized feedback and blended care in eHealth interventions (Kramer et al., 2014; Kroeze et al., 2017; Van Roekel et al., 2017). Several biopsychosocial factors may play a role in fatigue in chronic diseases, but interindividual differences are most likely large. The large interindividual differences in the outcomes of the personalized reports and advices found in the current study are in line with other studies describing large heterogeneity in networks between individuals, underscoring the importance of personalized medicine (Bos et al., 2018; Bosley et al., 2018; Molenaar, 2004). Nevertheless, no studies have yet been performed testing whether insights into a patient's network dynamics can significantly alter treatment decisions and patient outcomes (Wichers et al., 2017).

### 4.4 Strengths and limitations

A clear strength of our study is its relatively large sample size for a feasibility study, including adolescents with various chronic conditions. It shows the advantages of internet and smartphone-delivered EMA, combined with the potential of innovative ILD analyses (Hamaker and Wichers, 2017) to gain insight in fatigue. The possibilities the internet provides with controlled intensive and longitudinal data collection have high potential and lead to more complete and reliable datasets and better equidistant time series than EMA collected on paper. Together with the possibility to provide notifications on smartphones, which is suitable to the adolescent's lifestyle, this methodology is very promising for clinical practice, according to the involved adolescents. Another strength of this study is that we personalized the EMA prompts and questions, tailoring EMA to the daily life of the adolescent, which may lead to higher adherence (van Roekel et al., 2019).

Our study also had its limitations. First, we encountered several technical difficulties, especially at the start of the study, and consequently the compliance and motivation of some of the participants went down with a total compliance of 42%. Also, as a result of technical difficulties, compliance calculation was difficult, since duplicate data was generated for expired surveys for IPhone users at the start of the study. Considering this, we assume that when these difficulties will be resolved, the compliance may be even higher than found in this study. Second, there may have been a selection bias, since common reasons for not participating included an anticipated burden of filling out the EMA or unwillingness to be confronted with their fatigue. Nevertheless, there was no significant difference in fatigue questionnaire scores of the PROactive study between participants and non-participants. Third, female participants were overrepresented, so the results may be less generalizable to boys with chronic diseases. The mean age was relatively high for the included age range, but this was in line with our expectations, as fatigue is more prevalent among older adolescents (ter Wolbeek et al., 2006). Last, we did not calculate patterns in missing data on a group level. We therefore cannot exclude the possibility that missings were non-random, possibly leading to biased results. This is an important issue for future studies.

### 4.5 Suggestions for future research and clinical practice

For future research, assessment of the (long-term) effectiveness of internet and smartphone-based EMA and personalized advices based on single-subject dynamic network analyses reports in adolescents with chronic conditions is needed. Since EMA proved feasible in this population, other applications of EMA could be: assessing and comparing networks based on two EMA episodes assessed pre and post treatment, assessing whether a network changes during an intervention, or combining EMA with continuous feedback as an intervention in itself (Ecological Momentary Interventions; EMI) (Fisher et al., 2017; Snippe et al., 2017). The novelty of the ILD dynamic network analyses (RDSEM) make the interpretation of the data and the construction of the personalized report time consuming. Automation of these analyses and processes and automatic generation of a report will make EMA combined with ILD analyses and personalized reports more scalable for use in clinical practice. This also requires training of healthcare professionals how to interpret and explain a personalized report, as now this was mostly done by a clinical researcher.

## 5 Conclusion

Based on the compliance and quantitative results and the qualitative feedback presented in the current study, EMA combined with personalized advice based on a dynamic network analysis report (PROfeel), was demonstrated to be a feasible and useful method to improve health care for adolescents with chronic conditions suffering from fatigue. The way PROfeel renders personalized advices offers promising options for clinical practice, acknowledging that each person is unique, and enabling and motivating users to gain insight in and take steps towards treatment of fatigue, tailored to individual needs.

## Financial disclosure statement

The authors have no financial relationships relevant to this article to disclose.

## Funding

This research was partially supported by an eHealth grant of the Citrienfonds provided by the ‘Nederlandse Federatie Universitair Medische Centra (NFU)’ (PROfeel: an application for fatigued adolescents) and an unrestricted grant from 10.13039/100011022Vertex Pharmaceuticals (Circle of Care grant). Both funding sources had no involvement in the study design or the collection, analysis, and interpretation of data.

## Declaration of competing interest

The authors declare that they have no known competing financial interests or personal relationships that could have appeared to influence the work reported in this paper.

## Appendix A Supplementary data

Supplementary tables Image 9

## References

[bb0005] Armbrust Wineke (2015). Design and acceptance of Rheumates@Work, a combined internet-based and in person instruction model, an interactive, educational, and cognitive behavioral program for children with juvenile idiopathic arthritis. Pediatr. Rheumatol. Online J..

[bb0010] Armbrust Wineke (2016). Fatigue in patients with juvenile idiopathic arthritis: a systematic review of the literature. Semin. Arthritis Rheum..

[bb0015] Asparouhov T., Hamaker E.L., Muthen B. (2018). Dynamic structural equation models. Struct. Equ. Model. Multidiscip. J..

[bb0020] Bell Imogen H., Lim Michelle H., Rossell Susan L., Thomas Neil (2017). Ecological momentary assessment and intervention in the treatment of psychotic disorders: a systematic review. Psychiatr. Serv. (Wash. D.C.).

[bb0025] Bos F.M. (2018). Exploring the emotional dynamics of subclinically depressed individuals with and without anhedonia: an experience sampling study. J. Affect. Disord..

[bb0030] Bosley Hannah G., Fisher Aaron J., Taylor C. Barr (2018). Differential responses of positive affect, negative affect, and worry in CBT for generalized anxiety disorder: a person-specific analysis of symptom course during therapy. Psychother. Res..

[bb0040] Broderick Joan E., Vikingstad Gregory (2008). Frequent assessment of negative symptoms does not induce depressed mood. J. Clin. Psychol. Med. Settings.

[bb0045] Bromberg Maggie H. (2014). Self-reported pain and disease symptoms persist in juvenile idiopathic arthritis despite treatment advances: an electronic diary study. Arthritis Rheum..

[bb0050] Connelly, Mark et al. n.d.“Emotion regulation predicts pain and functioning in children with juvenile idiopathic arthritis: an electronic diary study.” J. Pediatr. Psychol. 37(1): 43–52. http://www.ncbi.nlm.nih.gov/pubmed/22037006 (July 21, 2016). 10.1093/jpepsy/jsr088 PMC3263771 22037006

[bb0055] Cruise Charles E. (1996). Reactive effects of diary self-assessment in chronic pain patients. Pain.

[bb0060] Deary V., Chalder T., Sharpe M. (2007). The cognitive Behavioural model of medically unexplained symptoms: a theoretical and empirical review. Clin. Psychol. Rev..

[bb0065] Dijk-Lokkart Van, Elisabeth M. (2019). Longitudinal development of cancer-related fatigue and physical activity in childhood cancer patients. Pediatr. Blood Cancer.

[bb0070] Dormann Christian, Griffin Mark A. (2015). Optimal time lags in panel studies. Psychol. Methods.

[bb0075] Ebner-Priemer Ulrich W., Trull Timothy J. (2009). Ecological momentary assessment of mood disorders and mood dysregulation. Psychol. Assess..

[bb0080] Elsbernd Abbey (2018). Cocreated smartphone app to improve the quality of life of adolescents and young adults with cancer (Kræftværket): protocol for a quantitative and qualitative evaluation. JMIR Res. Protoc..

[bb0085] Engel G.L. (1977). The need for a new medical model: a challenge for biomedicine. Science (New York, N.Y.).

[bb0090] Ethica Data (2019). Ethica.

[bb0095] Fisher Aaron J. (2017). Exploring the idiographic dynamics of mood and anxiety via network analysis. J. Abnorm. Psychol..

[bb0100] Frijns Tom, Keijsers Loes, Finkenauer Catrin (2020). Keeping secrets from parents: on galloping horses, prancing ponies and pink unicorns. Curr. Opin. Psychol..

[bb0105] Gordijn M. Suzanne (2011). Fatigue in children: reliability and validity of the Dutch PedsQLTM multidimensional fatigue scale. Quality of Life Research.

[bb0110] Hamaker Ellen L., Wichers Marieke (2017). No time like the present. Curr. Dir. Psychol. Sci..

[bb0115] Hofmann Stefan G., Hayes Steven C. (2019). The future of intervention science: process-based therapy. Clin. Psychol. Sci..

[bb0120] Holloway Immy, Wheeler Stephanie, Holloway Immy (2010). Qualitative Research in Nursing and Healthcare. https://books.google.nl/books/about/Qualitative_Research_in_Nursing_and_Heal.html?id=8AP3sCg1kdYC&redir_esc=y.

[bb0125] Houtveen Jan H., Sorbi Marjolijn J., Sommer Claudia (2013). Prodromal Functioning of Migraine Patients Relative to Their Interictal State–An Ecological Momentary Assessment Study.

[bb0130] Houtveen Jan H. (2015). The day-to-day concurrence of bodily complaints and affect in patients with severe somatoform disorder. Scand. J. Psychol..

[bb0135] Huang I.-C. (2013). The relationships between fatigue, quality of life, and family impact among children with special health care needs. J. Pediatr. Psychol..

[bb0140] Jóhannsdóttir Inga M.R. (2012). Increased prevalence of chronic fatigue among survivors of childhood cancers: a population-based study. Pediatr. Blood Cancer.

[bb0145] Kim Jiseon (2014). Symptoms and quality of life indicators among children with chronic medical conditions. Disabil. Health J..

[bb0150] Kramer Ingrid (2014). A therapeutic application of the experience sampling method in the treatment of depression: a randomized controlled trial. World Psychiatry.

[bb0155] Kratz Anna L., Murphy Susan L., Braley Tiffany J. (2017). Ecological momentary assessment of pain, fatigue, depressive, and cognitive symptoms reveals significant daily variability in multiple sclerosis. Arch. Phys. Med. Rehabil..

[bb0160] Kroeze Renske (2017). Personalized feedback on symptom dynamics of psychopathology: a proof-of-principle study. j. Person-Oriented Res..

[bb0165] Langeveld N.E. (2003). No excess fatigue in young adult survivors of childhood cancer. Eur. J. Cancer (Oxford, England: 1990).

[bb0170] Maurice-Stam Heleen (2019). Review about the impact of growing up with a chronic disease showed delays achieving psychosocial milestones. Acta Paediatr..

[bb0175] Menting Juliane (2018). Is fatigue a disease-specific or generic symptom in chronic medical conditions?. Health Psychol..

[bb0180] Molenaar Peter C.M. (2004). A manifesto on psychology as idiographic science: bringing the person Back into scientific psychology, this time forever. Meas. Interdiscip. Res. Perspect..

[bb0185] Myin-Germeys I. (2009). Experience sampling research in psychopathology: opening the black box of daily life. Psychol. Med..

[bb0190] Nap-van der Vlist Merel M. (2018). Prevalence of severe fatigue among adults with cystic fibrosis: a single center study. J. Cyst. Fibros..

[bb0195] (2019). Fatigue in childhood chronic disease. *Archives of Disease in Childhood*: archdischild-2019-316782.

[bb5000] Nijhof Sanne L., Bleijenberg Gijs, Uiterwaal Cuno S.P.M, Kimpen Jan L.L., van de Putte Elise M. (2011 Feb 19). Fatigue In Teenagers on the interNET-the FITNET Trial. A randomized clinical trial of web-based cognitive behavioural therapy for adolescents with chronic fatigue syndrome: study protocol. BMC Neurol..

[bb0205] Nijhof Sanne L. (2012). Effectiveness of internet-based cognitive behavioural treatment for adolescents with chronic fatigue syndrome (FITNET): a randomised controlled trial. Lancet.

[bb0200] Nijhof Linde N., Putte Elise M. van de, Wulffraat Nico M., Nijhof Sanne L. (2016 Jan). Prevalence of severe fatigue among adolescents with pediatric rheumatic diseases. Arthritis Care Res. (Hoboken).

[bb0210] Perrin James M., Bloom Sheila R., Gortmaker Steven L. (2007). The increase of childhood chronic conditions in the United States. JAMA.

[bb0215] Quanjer Philip H. (2012). Multi-ethnic reference values for spirometry for the 3-95-yr age range: the global lung function 2012 equations. Eur. Respir. J..

[bb0220] van Roekel Eeske, Keijsers Loes, Chung Joanne M. (2019). A review of current ambulatory assessment studies in adolescent samples and practical recommendations. J. Res. Adolesc..

[bb0225] Schoeppe Stephanie (2017). Apps to improve diet, physical activity and sedentary behaviour in children and adolescents: a review of quality, features and behaviour change techniques. Int. J. Behav. Nutr. Phys. Act..

[bb0230] Snippe Evelien (2017). The impact of treatments for depression on the dynamic network structure of mental states: two randomized controlled trials. Sci. Rep..

[bb0235] Stein Ruth E.K., Jessop Dorothy Jones (1989). What diagnosis does not tell: the case for a noncategorical approach to chronic illness in childhood. Soc. Sci. Med..

[bb0240] Stone Arthur A., Shiffman Saul (1994). Ecological momentary assessment (Ema) in behavioral medicine. Ann. Behav. Med..

[bb0245] Van Roekel Eeske (2017). An exploratory randomized controlled trial of personalized lifestyle advice and tandem skydives as a means to reduce anhedonia. Behav. Ther..

[bb0250] Verwey-Jonker Instituut (2019). Een Actueel Perspectief Op Kinderen En Jongeren Met Een Chronische Aandoening in Nederland.

[bb0255] Vijver Els Van de (2019). Fatigue in children and adolescents with inflammatory bowel disease. World J. Gastroenterol..

[bb0260] Voelkle Manuel C., Johan H.L. Oud, Oertzen Timo von, Lindenberger Ulman (2012). Maximum likelihood dynamic factor modeling for arbitrary *N* and *T* using SEM. Struct. Equ. Model. Multidiscip. J..

[bb0265] Wichers Marieke, Johanna T.W. Wigman, Bringmann Laura F., Jonge Peter de (2017). Mental disorders as networks: some cautionary reflections on a promising approach. Soc. Psychiatry Psychiatr. Epidemiol..

[bb0270] ter Wolbeek M., Lorenz J.P. van Doornen, Kavelaars Annemieke, Heijnen Cobi J. (2006). Severe fatigue in adolescents: a common phenomenon?. Pediatrics.

