# Supplementary material for: Internet and smartphone-based ecological momentary assessment and personalized advice (PROfeel) in adolescents with chronic conditions: A feasibility study
Source: Internet Interv. 2021 Apr 20;25:100395. doi: 10.1016/j.invent.2021.100395 (PMC8131314; doi:10.1016/j.invent.2021.100395)
Supplement: Supplementary file 1 — Supplementary tables [file mmc1.pdf]

Supplementary Table S1. Content of the EMA questionnaire

[always asked] **1 Is this the first time you fill out the survey after the night? [No, Yes]**

If 1 yes: **2 I slept last night for:** *[0-2 hours; 2-4 hours; 4-6 hours; 6-8 hours; 8-10 hours; 10-12 hours; longer than 12 hours]*

If 1 yes: **3 I felt rested when I woke up** *[visual analogue scale (VAS) 0-100 Not at all, a little, quite, good, very good]**

If 1 no: **4 For the last three hours I slept or lied resting during daytime (in bed or on the couch):** *[No Yes]*

If 1 no and 4 yes: **5 I slept or lied resting during daytime for:** *[0-1 hour; 1-2 hours; 2-3 hours; 3-4 hours; 4-5 hours; 5-6 hours; longer than 6 hours]*

If 1 no and 4 yes: **6 I felt rested afterwards** *[VAS 0-100 Not at all, a little, quite, good, very good]**

[always asked] **7 For the last three hours, the majority of the time I was:** *[At home; Somewhere else]*

[always asked] **8 For the last three hours, the majority of time I was doing things:** *[Alone, With others]*

[always asked] **9 For the last three hours, I was in touch with people who helped me, supported me or understood me** *[No, Yes]*

[always asked] **10 For the last three hours, I was in touch with people who bothered me, who I was in conflict with or who did not understand me** *[No, Yes]*

[always asked] **11 For the last three hours, I was physically:**

1. Inactive – Lying down, sleeping
2. A little bit inactive – sitting, reading, using the computer, tablet or smartphone
3. A little bit active – walking, shopping, doing chores
4. Very active – running, sporting

If 4 very active:

12 Amount of time that I was physically very active *[up to 15 minutes; 15 min – 30 min; 30 min – 1 hour; 1 hour – 2 hours; longer than 2 hours]*

[always asked] **13 For the last three hours, I was mentally:****

1. Inactive
2. A little bit inactive
3. A little bit active

4. Very active

If 4 very active:

14 Amount of time that I was mentally very active *[up to 15 minutes; 15 min – 30 min; 30 min – 1 hour; 1 hour – 2 hours; longer than 2 hours]*

[always asked] **15 The last three hours I was fatigued** *[VAS 0-100 from not at all to very much]**

[personalized depending on the baseline survey] **16-18 For the last three hours I was bothered by (personalized symptoms)** *[VAS 0-100 from not at all to very much]**

- 1, 2 or 3 personalized symptoms based on the baseline survey

[always asked] **19 For the last three hours, my symptoms restricted me in what I wanted to do (i.e. go to school, play sports, meet with friends)** *[VAS 0-100 from Not at all to very much]*

[personalized depending on the baseline survey] **20-22 In the last three hours, I was/had (personalized question(s), e.g. worried, anxious, proud)** *[VAS 0-100 from not at all to very]*

- 1, 2 or 3 psychosocial factors based on the baseline survey

[always asked] **23 Is today a school day?** *[No, Yes]*

If 23 yes : **24 Has your school day just finished?** *[No, Yes]*

If 23 and 24 yes: **25 I went to school today for:** *[I did not go; 1 hour; 2 hours; 3 hours; 4 hours; 5 hours; 6 hours; 7 hours; 8 hours or more]*

If 23 and 24 yes: **26 My classmates went to school today for:** *[There were no classes; 1 hour; 2 hours; 3 hours; 4 hours; 5 hours; 6 hours; 7 hours; 8 hours or more]****

27 Thank you for filling out this survey. If you want to (you do not have to) you can fill out an extra symptom or event here, if this was relevant for you the last three hours. Press submit to save your answers. *[non mandatory open text box]*

**On the VAS, a participant could slide to any number between 0-100, but there was an additional text visible when sliding back and forth that changed depending on the score.*

*** Mental activity was discussed with adolescents during the baseline survey and they often described it as working concentrated on a task or being alert during social interactions.*

**** We explained to the participants that we wanted to compare their participation to their total amount of hours. If participants had no classmates with the same schedule, they filled this out as the amount of hours they planned to go to school.*

Supplementary Table S2. Additional statistical background on the PROfeel analyses
Analyzing intensive longitudinal data with Residual Dynamic Structural Equation Modelling (RDSEM)<br>Dynamic analysis was needed to gain insight in the lagged and contemporaneous associations between fluctuations over the time in fatigue and associated factors. Individual dynamic networks were computed using a general modelling framework, referred to as Dynamic Structural Equation Modelling (DSEM) (Asparouhov, Hamaker, and Muthen 2018). DSEM can deal with several statistical challenges such as imputation of missing values, standardization of the estimates, and observations with unequal spaced time-intervals. A corresponding modelling framework for residuals correlated across time (Residual DSEM) was used to control for effects of the circadian cycle (e.g., participants normally report more fatigue in the evening), day-of-week cycles (of which weekend-effects are the most important), and non-stationary low-frequency trends.<br>We have applied a single-subject RDSEM, using MPLUS version 8.2. First, residuals were created corrected for low-frequency trends, weekend and time of day affects. Then a VAR(1) model is fitted on these residuals yielding standardized estimates for the contemporaneous and auto and cross-lagged temporal associations. The effects of the trends, and the time of day and weekend effects are visualized (as standardized estimates) in the RDSEM output.<br>In this RDSEM approach, latent variables are created, but they are not based on multiple indicators, Rather, these latent variables are residuals that are corrected for trends derived from the corresponding observed variables, weekend effects and time of day. You can consider our residuals as a filtered signal, for example, a residual without its own low-frequency trend. Then a VAR(1) is fitted on these residuals, which is a dynamic network interpretation comparable to the work of Bringmann et al., but more sophisticated (Bringmann et al., 2016). Therefore, the RDSEM results are referred to as individual dynamic (symptom) networks.<br><br>Reference: Bringmann, Laura F. et al. 2016. “Assessing Temporal Emotion Dynamics Using Networks.” *Assessment* 23(4): 425–35. https://pubmed.ncbi.nlm.nih.gov/27141038/ (December 22, 2020).
